# Rational and design of prophylactic cranial irradiation (PCI) and brain MRI surveillance versus brain MRI surveillance alone in patients with limited-stage small cell lung cancer achieving complete remission (CR) of tumor after chemoradiotherapy: a multicenter prospective randomized study

**DOI:** 10.1186/s12885-024-12123-x

**Published:** 2024-04-08

**Authors:** Mengyuan Chen, Runhua Li, Yue Kong, Lei Shi, Jing Wang, Yuezhen Wang, Yujin Xu, Yongling Ji, Xiao Hu

**Affiliations:** Zhejiang Cancer Hospital, Hangzhou Institute of Medicine (HIM), Chinese Academy of Sciences, Hangzhou, Zhejiang 310022 China

**Keywords:** Small cell lung cancer, Limited-stage, Prophylactic cranial irradiation, MRI surveillance

## Abstract

**Background:**

Prophylactic cranial irradiation (PCI) is part of standard care in limited-stage small cell lung cancer (SCLC) at present. As evidence from retrospective studies increases, the benefits of PCI for limited-stage SCLC are being challenged.

**Methods:**

A multicenter, prospective, randomized controlled study was designed. The key inclusion criteria were: histologically or cytologically confirmed small cell carcinoma, age ≥ 18 years, KPS ≥ 80, limited-stage is defined as tumor confined to one side of the chest including ipsilateral hilar, bilateral mediastinum and supraclavicular lymph nodes, patients have received definitive thoracic radiotherapy (regardless of the dose-fractionation of radiotherapy used) and chemotherapy, evaluated as complete remission (CR) of tumor 4–6 weeks after the completion of chemo-radiotherapy. Eligible patients will be randomly assigned to two arms: (1) PCI and brain MRI surveillance arm, receiving PCI (2.5 Gy qd to a total dose of 25 Gy in two weeks) followed by brain MRI surveillance once every three months for two years; (2) brain MRI surveillance alone arm, undergoing brain MRI surveillance once every three months for two years. The primary objective is to compare the 2-year brain metastasis-free survival (BMFS) rates between the two arms. Secondary objectives include 2-year overall survival (OS) rates, intra-cranial failure patterns, 2-year progression-free survival rates and neurotoxicity. In case of brain metastasis (BM) detect during follow-up, stereotactic radiosurgery (SRS) will be recommended if patients meet the eligibility criteria.

**Discussion:**

Based on our post-hoc analysis of a prospective study, we hypothesize that in limited-stage SCLC patients with CR after definitive chemoradiotherapy, and ruling out of BM by MRI, it would be feasible to use brain MRI surveillance and omit PCI in these patients. If BM is detected during follow-up, treatment with SRS or whole brain radiotherapy does not appear to have a detrimental effect on OS. Additionally, this approach may reduce potential neurotoxicity associated with PCI.

## Background

Small cell lung cancer (SCLC) accounts for approximately 15-20% of all cases of bronchopulmonary carcinoma. It is characterized by high malignancy, a tendency to early metastasis. At the time of diagnosis, about one-third of cases are of limited-stage [1]. The standard treatment is chemoradiotherapy for the majority of limited-stage SCLC patients [2].

A meta-analysis showed that prophylactic cranial irradiation (PCI) significantly reduce the incidence of BM by 25.3% compared to the control group (33.3% vs. 58.6%, *P* < 0.001), in patients who achieved complete remission (CR) after chemoradiotherapy, and it also improved the 3-year overall survival (OS) rate by 5.4% (20.7% vs. 15.3%, *P* = 0.01) [3]. Based on this study, PCI has been recommended for patients with limited-stage SCLC who have achieved a good response to chemoradiotherapy [2, 4].

However, the meta-analysis [3] has several limitations. The most important one is that no routine brain MRI was performed before PCI. A study conducted in the era of MRI demonstrated that 25% of newly diagnosed SCLC patients had brain metastasis (BM), and the cumulative incidence of BM after initial treatment could exceed 50% [5]. Our retrospective study showed that among all limited-stage SCLC patients who did not receive PCI and developed BM, 30.2% of them were detected before planned PCI with brain MRI [6]. Therefore, it can be speculated that some of the patients included in this meta-analysis actually received “treatment” rather than “prophylaxis”.

We have completed a prospective randomized study on the target volume of thoracic radiotherapy in limited-stage SCLC [7]. Our post-hoc analysis showed that, among 300 patients who received definitive chemoradiotherapy in this study, 134 (44.7%) achieved CR, 105 patients received PCI. All patients underwent baseline brain MRI, and 85.6% of them received brain MRI before PCI. The median follow-up time for the entire group was 22.3 months. Although PCI significantly reduced the incidence of BM (16.2% vs. 37.9%, *P* = 0.02), the median survival time for the PCI and the non-PCI arm were 30.2 months and 30.5 months, respectively, with 3-year OS rates of 39.9% and 43.0% (*P* = 0.93). PCI did not significantly improve the OS of patients who achieved CR after chemoradiotherapy. Among the 300 patients, 143 (47.7%) patients achieved partial remission (PR), of whom, 90 patients received PCI. Before PCI, 90.9% of patients received brain MRI. The median survival time for the PCI and the non-PCI arm were 27.4 months and 18.6 months, respectively, with 3-year OS rates of 42.6% and 17.4% (*P*<0.0001). PCI significantly improved the OS of patients who achieved PR after chemoradiotherapy (unpublished data).

Therefore, in the era of routine brain MRI surveillance, the role of PCI in limited-stage SCLC warrants further study. Based on above preliminary results, we speculate that PCI could be spared in patients who achieve CR after definitive chemoradiotherapy and instead, receive brain MRI surveillance. Even if BM is detected during follow-up, subsequent salvage treatment with SRS or whole-brain radiotherapy (WBRT) would not have a detrimental effect on OS.

## Methods

### Study design and objective

This is a prospective, randomized controlled study that includes patients with limited-stage SCLC who achieve CR after definitive chemoradiotherapy. Eligible patients will be randomly assigned to two arms:

1. Control arm: Patients receive PCI and regular brain MRI surveillance.
2. Study arm: Patients receive regular brain MRI surveillance alone.

If BM is detected during follow-up, SRS is recommended when appropriate.

The primary endpoint is to compare the 2-year brain metastasis -free survival (BMFS) rates between the two arms. Secondary endpoints include 2-year OS, intra-cranial failure patterns, 2-year progression-free survival (PFS), cognitive functions(Hopkins Verbal Learning Test will be used to assess cognitive function).

### Key eligibility criteria

Inclusion criteria:

1. Histologically or cytologically confirmed small cell carcinoma.
2. Age ≥ 18 years.
3. Karnofsky Performance Status (KPS) ≥ 80.
4. Limited-stage disease, defined as tumor confined to one side of the chest including ipsilateral lung, bilateral mediastinal lymph nodes, and bilateral supraclavicular lymph nodes (metastatic lymph nodes defined as short-axis diameter ≥ 1 cm or showing increased metabolic activity on PET-CT, or confirmed by mediastinoscopy / EBUS / TBNA biopsy). Pleural effusion thickness on chest CT is less than 1 cm (unless cytologically confirmed as malignant pleural effusion). Staging is determined according to the 8th edition of the AJCC staging system (2017), specifically stages I-IIIC without intrapulmonary metastasis.
5. Have received curative-intent thoracic radiotherapy and chemotherapy.
6. Assessment of treatment response within 4–6 weeks after completion of curative-intent thoracic chemoradiotherapy shows CR (evaluation includes contrast-enhanced chest and abdominal CT, contrast-enhanced brain MRI, whole-body bone scan, and lung cancer biomarkers such as NSE and ProGRP).
7. Willingness and ability to comply with the follow-up schedule.
8. Full understanding of the study and voluntary signing of an informed consent form.

Exclusion criteria:

1. History of malignant tumors in other parts of the body (previous or concurrent), excluding non-malignant melanoma, papillary carcinoma of the thyroid and cervical carcinoma in situ.
2. Patients who have undergone curative surgery (excluding biopsy).
3. Patients with a history of mental illness, in pregnancy or lactation.
4. Uncontrolled diabetes, hypertension, or severe active infections.
5. Manifested chronic central nervous system disorders.
6. Contraindications for brain MRI examination.
7. Other situations deemed unsuitable for enrollment by doctors in charge.

### Pre-treatment evaluation

Baseline staging include enhanced MRI of the brain, enhanced CT of the chest and upper abdomen, ultrasonography of the supraclavicular lymph nodes and bone scan. PET-CT is recommended, bone scan could be omitted if PET-CT is available. Laboratory tests include routine blood tests, routine liver and kidney function tests, lung cancer biomarker tests, electrocardiogram, echocardiography and pulmonary function test.

### Statistical analysis & sample size considerations

The study is designed as a prospective, randomized controlled non-inferiority trial. Based on previous study results, we hypothesize that the 2-year BMFS rate in the control group is 83%, and the 2-year BMFS in the study group is 68%, which is deemed acceptable as non-inferior. With a power of 80% and a one-sided significance level of 0.025, considering a shedding rate of 10%, 110 patients will be required in each group.

## Radiotherapy

### Positioning and CT/MRI Simulation

All patients will be placed in supine position with thermoplastic mask for whole-brain immobilization. Contrast-enhanced CT simulations are recommended. The slice thickness of the scan should be ≤ 5 mm, and the scanning range should extend from 2 cm above the skull to the lower margin of the 7th cervical vertebra, including the entire cranial and neck. When PCI with hippocampal avoidance is required, a MRI simulation of the brain is strongly recommended and the thickness of the brain MRI scanning should be 1 mm.

### Definition of radiation target volume

The entire brain is contoured as the clinical target volume (CTV), and the CTV is expanded by 0.3 cm to create the planning target volume (PTV) which is named PTV-Brain. The PTV of hippocampal avoidance PCI is named PTV-HA. PTV-HA is PTV-Brain minus hippocampi. Critical organs at risk (OAR) include the bilateral lenses and eyeballs. For patients who are eligible for brain MRI simulation, bilateral hippocampi should be contoured.

### Radiation dose and planning evaluation

The prescribed dose for PTV is 2.5 Gy per fraction, once daily, 5 days a week for two weeks. The minimum requirement for the radiation technique is three-dimensional conformal radiotherapy. If PCI with hippocampal avoidance is required, the minimum requirement for irradiation method is stated to be IMRT. Helical tomotherapy is recommended if available.After completing the radiation treatment planning, the dose distribution in the target volume and the dose to OAR are evaluated. The dose-volume histogram (DVH) is used as a fundamental tool, and the dose distribution in the PTV and OAR is assessed based on the distribution of dose curves in three-dimensional space. The prescribed dose is determined based on the dose received by 95% of the PTV, with dose uniformity ranging from 94 to 106%. The dose constraints for OAR are as follows: lenses: Dmax < 9 Gy, Dmean < 5 Gy; hippocampi: Dmax < 16 Gy, Dmean < 9 Gy.

### Toxicity evaluation

Treatment related toxicities will be evaluated in accordance with Common Terminology Criteria for Adverse Events (CTCAE) 5.0 criteria. (1) Complete blood counts and physical examinations will be performed at least once a week during radiation therapy. (2) Adverse events related to the nervous system will be documented at least once a week during radiation therapy and during follow-up. Hopkins Verbal Learning Test will be used to assess cognitive function.

### Tumor response evaluation criteria and definition of survival time

Tumor response will be evaluated according to Response Evaluation Criteria in Solid Tumors (RECIST) 1.1 criteria.

Brain metastasis-free survival (BMFS) is defined as the time from the start of treatment to BM or death from any cause. Progression-free survival (PFS) is defined as the time from the start of treatment to disease progression or death from any cause. Overall survival (OS) is defined as the time from the start of treatment to death from any cause.

### Follow-up

All enrolled patients will be followed up starting from the start of PCI and continuing for 2 years or until death. Follow-up will be conducted once every three months. At each follow-up, patients routinely received examination of brain enhanced MRI, chest and abdomen enhanced CT, and lung tumor markers. After two years, the frequency of follow-up is left to the discretion of the doctor in charge.

### Radiotherapy for brain metastasis

For patients with ≤ 5 intracranial metastases and a maximum diameter of ≤ 3 cm for a single metastasis, SRS or Cyber Knife radiotherapy will be recommended. Otherwise, WBRT will be administered, especially for patients who have not yet received PCI. For patients who have received PCI and developed BM, SRS or Cyber Knife will be recommended.

## Discussion

SCLC tends to develop early distant metastasis. Brain is a common site of metastasis in SCLC patients, with approximately 25% of patients present with brain metastases at the time of initial diagnosis [5]. Due to the presence of the blood-brain barrier, conventional chemotherapy drugs have limited efficacy in penetrating brain tissue, making it a potential “sanctuary” for small brain metastatic lesions. Therefore, PCI does not truly “prevent” the development of BM, it plays a role of eliminating potentially but undetectable small metastasis in brain.

With the advances in comprehensive treatment, the prognosis of limited-stage SCLC patients has improved, the probability of BM has also increased correspondingly. Among patients who achieved CR after treatment, the incidence of BM within 2 years was 67%, with the brain being the first site of metastasis in 45% of cases [8].

A meta-analysis demonstrated that in patients who achieved a complete response (CR) after chemoradiotherapy, PCI could significantly reduce the incidence of BM by 25.3% (*P* < 0.001) compared to the control group. Additionally, it was associated with a 5.4% improvement in 3-year overall survival rate (*P* = 0.01) [3].

However, due to the limitations of the meta-analysis, and with the availability of MRI for regular surveillance of BM, the role of PCI in improving OS in SCLC patients is being challenged.

A prospective randomized study conducted by EORTC demonstrated that extensive-stage SCLC patients who received PCI had significantly better outcomes compared to the observation group (1-year incidence of symptomatic BM: 14.6% vs. 40.4%; median survival time: 6.7 months vs. 5.4 months; 1-year overall survival rate: 27.1% vs. 13.3%) [9]. However, one of the major limitations of this study was the absence of pre-PCI brain MRI. In contrast, a phase III randomized study showed that in extensive-stage SCLC patients who have ruled out BM with MRI, PCI did not have a positive impact on OS, compared to the observation group [10].

Traditionally, WBRT is recommended for SCLC patients with BM. A recent large cohort study [11] compared the outcomes of SCLC patients with BM who received SRS or WBRT. The results showed that those treated with SRS had a median survival time of 8.5 months and a time to central nervous system progression (TTCP) of 8.1 months, while patients with a solitary BM treated with SRS had a median survival time of 11.0 months, and those treated with WBRT had a median survival time of 5.2 months. Although WBRT improved TTCP, it did not improve OS. Furthermore, after adjusting for prognostic factors, the OS results favored SRS.

Another study showed that in limited-stage SCLC patients who underwent MRI surveillance after definitive chemoradiotherapy, the occurrence of BM and survival rates did not significantly differ between patients who received SRS for detected BM and those who received PCI [12]. The survival benefit was attributed at least partially to SRS, reducing the contribution of PCI to survival. Although PCI is still recommended based on previous studies, these benefits may be diminished if MRI and SRS are available for diagnosis and treatment.

These findings challenge the traditional approach of using WBRT or PCI for all SCLC patients with BM and suggest that SRS guided by MRI surveillance could be a viable alternative in selected cases. However, it is important to consider individual patient factors, tumor characteristics, and treatment goals when determining the most appropriate management strategy.

Pezzi et al. [13] reported that in patients with limited-stage SCLC who had BM excluded by MRI were matched using a propensity score, although the 3-year incidence of BM was higher in the non-PCI group than in the PCI group, the difference was not statistically significant (20.4% vs. 11.2%, *P* = 0.10). Also, whether PCI was performed or not did not affect the overall survival (HR: 0.84, 95% CI: 0.604–1.180, *P* = 0.32).

Similarly, Qi et al. [14] conducted a retrospective matched analysis of 150 patients with limited-stage SCLC. The results showed a significantly lower 3-year cumulative incidence of BM in the PCI group compared to the non-PCI group (14.7% vs. 22.7%, *P* = 0.007), but there was no significant difference in median survival time between the two groups (35 months vs. 28 months, *P* = 0.128).

Of note, PCI potentially leads to acute and late neuro-toxicities. A pooled analysis of the RTOG 0212 and 0214 showed that patients who received PCI had reduced cognitive function as measured by the Hopkins Verbal Learning Test at the 6-month and 12-month follow-up, compared to baseline (*P* = 0.002). Patient-reported cognitive decline was even three times higher (*P* < 0.0001) [15]. A survey indicated that 38% of limited-stage SCLC patients who did not receive PCI had concerns about the side effects [16].

At present, there is no prospective randomized controlled study on PCI versus regular MRI follow-up after definitive radio-chemotherapy for limited-stage SCLC available. However, several similar studies are underway. SWOG S1827 [17] is a phase III prospective randomized controlled study that aims to enroll patients with limited-stage SCLC who have received radical therapy and patients with extensive-stage SCLC who have responded to systemic therapy. The study will randomize patients into two groups: one group will receive PCI plus regular brain MRI follow-up, while the other group will receive only regular brain MRI follow-up. The primary objective of the study is to compare the two-year survival rates between the two groups. The PRIMA Lung Study [18] is conducted by EORTC, has similar study design, aims to investigate whether brain MRI surveillance alone is non-inferior in terms of OS compare to PCI followed by brain MRI surveillance in both limited and extensive-stage SCLC patients. Another Chinese study [19] recruits and randomize limited-stage SCLC patients who achieve remission after first-line chemoradiotherapy to PCI or MRI surveillance. The primary end point is OS at two years.

In conclusion, with the widespread use of brain MRI, the favorable prognosis of SRS for treating SCLC BM, and the insights gained from the Japanese prospective study on PCI in extensive-stage SCLC, as well as the emphasis on the quality of life of long-term survivors, it is worthwhile to conduct a prospective randomized study comparing active brain MRI surveillance alone with PCI after chemoradiotherapy for limited-stage SCLC. The results of this study are highly likely to change current clinical practices.

## Data Availability

Not applicable.
